# Factors that influence fatigue status in patients with severe rheumatoid arthritis (RA) and good disease outcome following 6 months of TNF inhibitor therapy: a comparative analysis

**DOI:** 10.1007/s10067-015-3088-6

**Published:** 2015-10-09

**Authors:** Patricia Minnock, Douglas J. Veale, Barry Bresnihan, Oliver FitzGerald, Gabrielle McKee

**Affiliations:** Rheumatic and Musculoskeletal Disease Unit, Our Lady’s Hospice & Care Services, Harold’s Cross, Dublin 6, Ireland; Bone and Joint Unit, St Vincent’s University Hospital, Dublin, Ireland; School of Medicine and Medical Science and Conway Institute of Biomolecular and Biomedical Research, University College Dublin, Dublin, Ireland; School of Nursing & Midwifery, Trinity College Dublin, Dublin, Ireland

**Keywords:** Disease outcome, Fatigue, Pain, Rheumatoid arthritis, Self-efficacy

## Abstract

The objective of the present study is to determine the factors associated with persistent fatigue in patients with severe rheumatoid arthritis (RA) and good disease response to 6 months of tumour necrosis factor inhibitor therapy. Eligible patients with either persistent (PF) or no fatigue (NF) were compared. Using validated questionnaires and bivariate analysis, this cross-sectional survey explored if clinical characteristics, pain, self-efficacy, sleep and mood/depression differed between groups. Patients with PF (PF; NF) (*n* = 28; 28) reported significantly more overall pain (11.3 ± 9.4 (0–33); 6.9 ± 8.9 (0–33)), more recent and current pain intensity (41.4 ± 26.6 (0–80) 24.4 ± 26.6 (0–100) and depression (11.8 ± 7.5 (1–35); 8.2 ± 6.6 (0–26)), than the NF group. There was no significant difference between groups in self-efficacy and both groups experienced poor sleep quality (Pittsburgh Sleep Quality Index >5). Despite having good disease response, the PF group had significantly higher rheumatoid factor incidence, disease activity score-28, early morning stiffness duration and lower incidence of ever-failing disease-modifying anti-rheumatic drugs than the NF group. These findings enhance the fatigue literature in patients with RA prescribed tumour necrosis factor (TNF) inhibition therapy, identifying the potentially modifiable factors of pain and depression, previously demonstrated to be strongly associated with fatigue in non-biologic populations. In addition, this study highlights the association between persistent fatigue and an on-going state of low disease activity. This infers that more judicious disease management could minimise the symptom burden of pain and depression and consequentially fatigue.

Fatigue is recognised as an important symptom in rheumatoid arthritis (RA) by patients and clinicians [1–3]. While not yet defined in clear terms, its multidimensional nature has been identified [4, 5]. Studies have shown that the causes and consequences of fatigue in RA can be attributed to illness-related aspects, physical functioning, cognitive/emotional functioning and social aspects [6]. It is acknowledged that RA fatigue likely results from a complex interplay between these factors [5, 7] and requires further study to determine causation and best management [3, 6, 7].

Debate around to what degree fatigue is a primary or secondary symptom of the autoimmune inflammatory process [8, 9] has occurred in parallel with the positive impact of biologic drug therapies on disease management and patient-reported outcome [3, 8, 10], the Outcome MEasurement in Rheumatology Clinical Trials (OMERACT) patient perspective initiative [1, 11, 12] and the international endorsement of fatigue as a patient-centred outcome for inclusion in all patient studies [2]. Despite this elevated status, from patients’ perspectives, fatigue remains unmanageable and professional support remains rare [13].

The overarching aim of this study was to contribute to the rational development of appropriate prevention and management strategies for fatigue. This is contingent on identifying its drivers. Reasons for minimal insight into fatigue to date include (i) a lack of data from longitudinal observational studies exclusive to patients with RA, (ii) studies that were either confined to patients with only mild to moderately active disease [14] or (iii) studies that excluded populations from biologic registers on the basis of disease severity [9]. While researchers have identified key contributory factors, such as pain, sleep, self-efficacy and mood [9, 15], none has reported investigating the relevance of these factors in patients in different disease states of activity with differing fatigue levels, especially in the context of modern biological therapies [3]. Defining persistent fatigue as patient-reported presence of moderate or greater fatigue, the purpose of this study was to examine the factors associated with persistent fatigue in patients with established RA despite demonstrating a good disease response to 6 months of tumour necrosis factor (TNF) inhibitor therapy.

## Methods

### Study design and patient population

This cross-sectional single-site study used an observational, naturalistic comparative design. The procedures followed in this study are in accordance with the ethical standards of the relevant Ethics and Medical Research Committees and with the Helsinki Declaration of 1975, as revised in 1983. The study population was patients with (1) confirmed RA [16], (2) minimal age 18 years and (3) who had demonstrated a moderate to good disease response following 6 months of TNF inhibitor treatment for moderate to high disease activity [8], according to the disease activity score-28 (DAS28)-based European Union of Leagues against Rheumatism (EULAR) Response Criteria (ERC) [17]. Patients were excluded from the study if they had a primary diagnosis of fibromyalgia syndrome or chronic fatigue syndrome.

The DAS28 is a standardised, composite outcome measure of disease activity calculated using a four variable formula (DAS28-4 (variable) (C-reactive protein (CRP)) = 0.56 ∗ √ (TJC28, 28 joint count for tenderness) + 0.28 ∗ √(SJC28, 28 joint count for swelling) + 0.36 ∗ ln (CRP) + 1) + 0.014 ∗ (GH, patient global health assessment on a 0–10 numeric rating scale) + 0.96. The DAS28 is a continuous scale ranging from 0 to 9.4, indicating level of RA disease activity. DAS28 >5.1 reflects high disease activity, 5.1–3.2 reflects moderate disease activity, and <3.2 indicates low disease activity. Remission is achieved when a DAS28 score <2.6 is obtained. The ERC calculates both the degree of reduction in DAS28 as well as current DAS28 so as to classify individual patients into three categories of disease response: good (DAS28 improvement of >1.2), moderate (DAS28 improvement of >0.6–≤1.2) and non-response (DAS28 improvement of <0.6) [17]. Using the ERC, patients who had achieved at least a moderate improvement in their DAS28 in response to 6-month TNF inhibition therapy were identified.

To meet the analysis needs of this study, assuming a large effect size (0.8), alpha of 0.05 and power of 0.80, a sample size of 54 was required, with a minimum of 27 in each group [18]. Given the small sample size, the exact *p* value with the Z approximation test was reported; *p* values <0.05 were regarded as statistically significant. Holm’s sequential Bonferroni post hoc adjustment was used in the comparison tests to prevent type 1 error [19].

### Data collection

Purposive consecutive sampling was used to recruit patients meeting the inclusion criteria over a period of 18 months. Sociodemographic details, clinical characteristics and clinical assessments of disease activity using the ACR core set of outcome measures (28-swollen joint count, 28-tender joint count, pain, patient global health assessment, functional assessment using the Health Assessment Questionnaire Disability Index (HAQ-DI) and biochemical acute phase reactant measure, C-reactive protein (CRP)) [20], along with fatigue levels, were derived from the case notes. In all scales, higher scores indicated worst outcome. Following telephone contact to inform patients of the study, questionnaires to assess pain, self-efficacy, sleep, mood and depression were posted to eligible participants enclosed with an invitation to partake in the study, a consent form and a prepaid addressed envelope. All persons gave their informed consent prior to their inclusion in the study.

### Data collection tools

The sociodemographic and clinical characteristics derived from the case notes were gender, age, educational background, current smoking status, disease duration, rheumatoid factor (RF), haemoglobin level, disease-modifying anti-rheumatic drug (DMARD) history and early morning stiffness (EMS) duration in minutes. DAS28 and the derived ERC were the indices used to identify the study population.

#### Fatigue

Fatigue was measured using a standard validated five-point verbal scale recording fatigue over the last week. This scale has previously demonstrated good validity and sensitivity in patients with RA [4]. Patients indicated their level of fatigue as being the following: none, mild, moderate, severe and very severe. These data were used to differentiate between those with and without persistent fatigue,

#### Pain

Pain was assessed using the multidimensional Short-Form McGill Pain Questionnaire (SF-MPQ) [21]. It contains five separate pain scores. The first three (i) a sensory descriptor score of the pain experience, (ii) an affective descriptor score of the pain experience and (iii) a composite total descriptor score for pain over the past week were rated on a four-point pain intensity scale (none (0)–severe (3). The remaining two captured (iv) overall past week pain intensity using a 100-mm visual analogue scale (VAS) and (v) current pain intensity captured verbally using five descriptive terms (0 = ‘no pain’ to 5 = ‘excruciating’ pain). In this study, internal consistency (Cronbach’s *α* 0.72) was found to be consistent with previous values shown in RA and fibromyalgia populations (Cronbach’s *α* 0.73 to 0.89) [22].

#### Self-efficacy

Patient self-efficacy (SE) was measured using the Arthritis Self-Efficacy Scales (ASES), incorporating three subscales, which is extensively used in RA studies [23]. The ASES includes 20 items scored separately on a Likert scale of 1 (very uncertain) to 10 (very certain) from which the overall mean of the subscale items is derived and higher scores indicate greater confidence or SE. The three subscales are as follows: pain (five items), functioning (nine items) and other symptoms of SE (six items). The derived internal consistency (Cronbach’s *α* 0.79–0.84) was consistent with previously reported values (Cronbach’s *α* 0.76 to 0.89) [24].

#### Sleep

The Pittsburgh Sleep Quality Index (PSQI), a 19-item self-rated questionnaire was used to measure seven different components of sleep, duration, quality, number of arousals, efficacy, disturbances, use of sleep medications and daytime dysfunction during the previous month. Its suitability for use in patients with RA is documented [25]. The PSQI has reported high construct validity [26], sensitivity and specificity. Scores range from 0 to 25; a total score of >5 is indicative of sleep disturbances. In this study, internal consistency among the seven component scores was somewhat lower (Cronbach’s *α* 0.65) than that reported (Cronbach’s *α* 0.83) in generic studies [26].

#### Mood

Two separate scales were used to assess different aspects of mood and depression. The Profile of Mood States (POMS) short form, a widely used tool to assess transient and distinct mood states, has been used in a variety of RA fatigue studies [27]. This 37-item scale can be scored into five subscales (tension-anxiety, depression-dejection, fatigue-inertia, confusion-bewilderment and vigour). Each item is rated from 0—not at all to 4—extremely. Its discriminant validity is supported by being consistently more highly related to corresponding mood measures (for example, sad and depressed) (mean *r* = 66.6), than non-corresponding mood scales (for example, vigour versus inertia) (mean *r* = 49.5), when compared with other scales [28]. Internal consistency in this study using Cronbach’s *α* ranged from 0.54 to 0.86 across the six items.

The Beck Depression Inventory II (BDI-II), an extensively tested and validated questionnaire, comprises a series of 21 questions developed to measure the intensity, severity and depth of depression. Each question assesses a specific symptom, 13 items assess psychological symptoms, and 8 items assess physical symptoms. Each item uses a four-point scale (0 to 3), and total score range is 0–63. A score of 0–13 is considered minimal, 14–19 mild, 20–28 moderate and 29–63 severe. Its appropriateness as a screening tool for depression in patients with RA has been demonstrated [29]. It has a high construct validity (Cronbach’s *α* = (0.80) and discriminates between depressed and non-depressed groups [30]. Permission to use all scales was secured.

#### Analysis

The sample was subdivided into two groups for comparative purposes. Those who self-reported moderate or greater fatigue at time of recruitment were labelled the persistent fatigue group while those who reported mild or no fatigue were labelled the no-fatigue group. To enhance sample homogeneity, the two groups were matched approximately for gender, age and disease duration. Analysis was conducted using the statistical package SPSS 16.0 for Windows. Numbers and percentage counts were used to report nominal data (education/smoking status/RF); mean and standard deviations were reported on the normally distributed variables (DAS28) and medians and range for skewed data such as haemoglobin (HB) and early morning stiffness (EMS) duration. Inferential statistics to test for differences between subgroup means included chi-square, independent sample *t* tests and Mann-Whitney *U* test as appropriate. The significance level was originally set at 0.05 and then subjected to a Holm’s sequential Bonferroni adjustment as multiple tests which are indicated at the bottom of the relevant table.

## Results

### Patient demographic and clinical details

All eligible patients who were invited to take part in the study accepted (*n* = 64), and all patients gave their informed consent prior to their inclusion in the study. Of these 64 patients, 47 % (*n* = 28) qualified as the persistent fatigue group. The other 53 % (*n* = 36) of patients demonstrated a good fatigue outcome, qualifying as the no-fatigue group, from which 28 patients were purposively selected based on age, gender and disease duration.

The majority of the patients were female. There was no significant difference between the groups’ smoking status, educational background or HB. Significantly higher RF-positive status, EMS duration and mean DAS28 and a significantly lower incident of ever failed a disease-modifying anti-rheumatic drug (DMARD) were seen in the persistent fatigue group. While the difference between groups for the HAQ-DI was not statistically significant, it did exceed the known minimal clinical important difference [31] (Table 1).

**Table 1:** Sociodemographic and clinical characteristics of patients with good disease outcome and either persistent or no fatigue

Patients characteristics		Persistent fatigue	No fatigue	*p* value
*n* (study, control)		*n* (valid %)	*n* (valid %)	
Female gender^a^ (*n* = 28, 28)		22 (79)	23 (82)	≤0.213
Age (mean ± SD (range), years)^b^		58 ± 11 (26–77)	58 ± 11 (23–81)	≤0.716
(*n* = 28,28)				
Disease duration (mean ± SD (range), years)^b^		14 ± 11 (0–36)	14 ± 12 (0–39)	≤0.949
(*n* = 28, 28)				
Smoking status^a^	Current	9 (32)	7 (29)	≤0.139
(*n* = 27, 24)				
Educational background^a^	Primary	7 (25)	4 (20)	
(*n* = 26, 21)	Secondary	12 (43)	7 (35)	
	Third level	6 (21)	9 (45)	
Rheumatoid factor^a^	Positive	15 (54)	4 (14)^a^	=0.006*
(*n* = 28, 28)	Negative	13 (46)	23 (82)	
Ever-failed DMARD^a^	Yes	18 (64)	24 (86)^a^	=0.036*
(*n* = 28, 27)	No	10 (36)	3 (11)	
Ever-failed biologic	No	0	0	
(*n* = 28, 28)				
Haemoglobin levels (median (range), g/dl)				
(*n* = 22, 13)	6 months	13 (9–15)	13.4 (9–17)	≤0.452
HAQ-DI (mean ± SD)				
(*n* = 26, 16)	6 months	1.11 ± (0.62)	0.76 ± (0.55)	=0.07
Early morning stiffness duration (median, min)^c^				
(*n* = 27, 26)	6 months	10 (0–180)	10 (0–30)	=0.001*
Swollen joint count (mean ± SD)				
(*n* = 28, 16)	6 months	2.2 ± (2.9)	1.0 ± (1.7)	<0.135
Tender joint count (mean ± SD)				
(*n* = 28, 16)	6 months	3.2 ± (4.7)	1.0 ± (2.3)	<0.028*
Pain (mean ± SD)				
(*n* = 28, 16)	6 months	4.4 ± (2.2)	2.3 ± (1.0)	<0.001*
Patient global health (mean ± SD)				
(*n* = 28, 16)	6 months	4.9 ± (2.2)	2.3 ± (1.0)	<0.0001*
C-reactive protein (mean ± SD)^c^				
(*n* = 28, 16)		4 (0–42)	4 (0–27)	<0.718
C-reactive protein *n* (%)	6 months			
<2		2 (7.1)	1 (5.9)	
<10		21 (75.0)	12 (70.6)	
>10		5 (17.9)	4 (17.9)	
DAS-28 (mean ± SD, min-max)^c^				
(*n* = 28, 16)	6 months	3.3 ± (1.1)		
(1.7–5.8)	2.4 ± (0.7)			
(1.2–4.4)	=0.002*			
DAS-28	6 months			
>3.2		15 (62.5)	1 (37.5)	
<2.7		9 (7.1)	13 (92.9)	

*HAQ-DI* Health Assessment Questionnaire Disability Index, *DMARD* disease-modifying anti-rheumatic drugs

*Significance at the <0.05

^a^Chi-square test.

^b^Independent sample *t* test

^c^Mann-Whitney *U* test

A significant difference between fatigue groups was demonstrated in total pain experienced during the previous week (*u* = 0 241, *p* = 0.021). Sensory (*u* = 0 251, *p* = 0.02), pain intensity VAS (*u* = 0 223, *p* = 0.023) and current pain intensity subscales (*u* = 0 231, *p* = 0.009) were also significant (Table 2).

**Table 2:** Comparative analysis of pain experience in patients with good disease outcome and either persistent or no fatigue (Mann-Whitney *U* test)

Short-Form McGill Pain Questionnaire (SF-MPQ) *n* (study, control) (scale range)		Persistent fatigue	No fatigue		
	Descriptors (during last week)	Mean ± standard deviation (range)		*p* value	Level of significance^a^
	Mean sensory (*n* = 27, 28) (0–33)	8.0 ± 6.4 (0–24)	5.2 ± 6.5 (0–23)	0.02*	0.013
	Mean affective (*n* = 27, 28) (0–12)	2.7 ± 3.3 (0–12)	1.8 ± 2.8 (0–10)	0.235	0.05
A:	Total descriptor score (*n* = 27, 28) (0–45)	11.3 ± 9.4 (0–33)	6.9 ± 8.9 (0–33)	0.021*	0.016
B:	Past week: VAS pain intensity (*n* = 27, 26) (0–100 mm)	41.4 ± 26.6 (0–80)	24.4 ± 26.6 (0–100)	0.023*	0.025
C:	Current: pain intensity *n* (valid %) (*n* = 27, 28)				
	No pain	3 (11)	9 (32)	0.009*	0.01
	Mild	6 (22)	11 (39)		
	Discomforting	13 (48)	5 (18)		
	Distressing	3 (11)	2 (7)		
	Horrible	2 (7)	0		
	Excruciating	0	1 (3)		

*Significant

^a^Level of significance as set using Holm’s sequential Bonferroni adjustment

The three subscales for SE pain, functioning and other symptoms did not differ significantly between fatigue groups (Table 3). There was no significant difference in the total sleep score or the sleep subscales between groups (Table 4). There was also no difference between groups in relation to use of sleep medications. The sleep score greater than 5 recorded in both groups was clinically indicative of poor sleep quality.

**Table 3:** Comparative analysis of arthritis self-efficacy in patients with good disease outcome and either persistent or no fatigue (Mann-Whitney *U* test)

Subscales (range 1–10) (*n* = 28)		Persistent fatigue	No fatigue		
		Mean ± SD		*p* value	Significance level^a^
Self-efficacy pain subscale: How certain are you that you can…					
1	Decrease your pain quite a bit?	5.9 ± 2.0	6.1 ± 3.0		
2	Continue most of your daily activities?	6.5 ± 2.5	6.9 ± 2.9		
3	Keep arthritis pain from interfering with your sleep?	6.1 ± 3.0	6.7 ± 2.8		
4	Make small/moderate reduction in pain?	5.5 ± 2.7	4.5 ± 2.9		
5	Make large reduction in your arthritis pain by using methods other than taking extra medication?	4.3 ± 2.7	4.8 ± 3.2		
Mean pain		5.6 ± 1.9	6.1 ± 2.3	0.359	0.025
Self-efficacy function subscale: As of now how certain are you that you can					
1	Walk 100 ft on flat ground in 20 s?	6.7 ± 3.3	7.6 ± 3.1		
2	Walk ten steps downstairs in 7 s?	6.3 ± 3.3	6.7 ± 3.1		
3	Get out of armless chair quickly without using your hands for support?	5.7 ± 3.4	6.7 ± 3.1		
4	Button and unbutton three medium-size buttons in a row in 12 s?	6.8 ± 3.1	7.7 ± 2.4		
5	Cut two bite-size pieces of meat with a knife and fork in 8 s?	6.8 ± 3.2	7.5 ± 2.7		
6	Turn an outdoor tap all the way on and off?	6.6 ± 2.9	6.8 ± 3.1		
7	Scratch your upper back with both your right and left hands?	5.1 ± 3.3	5.8 ± 3.3		
8	Get in and out the passenger side of car without assistance from another person and without physical aids?	7.2 ± 25	7.7 ± 2.9		
9	Put on long-sleeved front-opening shirt or blouse (without buttoning) in 8 s?	7.2 ± 2.9	8.2 ± 2.5		
Mean function		6.7 ± 2.4	7.1 ± 2.5	0.413	0.05
Self-efficacy other symptom subscale: How certain are you that you can					
1	Control your fatigue?	4.9 ± 2.4	6.1 ± 2.3		
2	Regulate your activity so as to be active without aggravating your joints?	6.2 ± 2.6	7.0 ± 2.4		
3	Do something to help yourself feel better if feeing blue?	6.1 ± 2.7	7.4 ± 2.7		
4	Manage arthritis pain during daily activities?	6.4 ± 2.8	7.2 ± 2.5		
5	Manage arthritis symptoms so that you can do the things you enjoy doing?	5.7 ± 2.7	7.0 ± 2.8		
6	Deal with frustration of arthritis?	5.9 ± 2.8	7.1 ± 2.8		
Mean other symptom		5.8 ± 2.4	7.2 ± 2.2	0.022	0.017

^a^Level of significance as set post-Holm’s sequential Bonferroni adjustment

**Table 4:** Pittsburgh Sleep Quality Index (PSQI): comparative analysis of sleep quality in patients with good disease outcome and either persistent or no fatigue (Mann-Whitney *U* test).

Pittsburgh Sleep Quality Index (range)	Persistent fatigue *n* = 28	No fatigue *n* = 28	
	Mean ± SD (range) (0–3)		*p* value
Sleep duration (0–3)	1.1 ± 1.1 (0–3)	0.6 ± 0.9 (0–3)	0.061
Sleep disturbance (0–3)	1.5 ± 1.0 (0–3)	1.5 ± 0.8 (0–3)	0.901
Sleep latency (0–3)	1.4 ± 1.1 (0–3)	1.3 ± 1.0 (0–3)	0.856
Daytime dysfunction(0–3)	1.2 ± 1.0 (0–3)	1.0 ± 0.8 (0–3)	0.805
Sleep efficiency (0–3)	1.4 ± 1.2 (0–3)	1.2 ± 1.1 (0–3)	0.808
Overall sleep quality (0–3)	1.2 ± 1.2 (0–3)	1.0 ± 0.9 (0–3)	0.832
Sleep medications (0–3)	0.7 ± 1.2 (0–3)	0.5 ± 0.9 (0–3)	0.966
Total PSQI (0–21)	8.3 ± 3.6 (4–16)	7.3 ± 4.5 (0–18)	0.319

The mean BDI-II Score ± SD (range) of 11.8 ± 7.5 (1–35) for the persistent fatigue group was shown to be statistically different from that of the no-fatigue group 8.2 ± 6.6 (0–26), *U* = 265 and exact *p* = 0.037 (two-tailed). Total scores on the BDI-II were in what is considered to be the mild (0–13) to moderate range (14–19) for depression. Mean mood (POMS) total scores or subscales did not differ significantly between groups (Table 5).

**Table 5:** Comparative analysis of mood and depression in patients with good disease outcome and either persistent or no fatigue (Mann-Whitney *U* test)

Profile of mood states (range)	Persistent fatigue	No fatigue	
	Mean (SD), range *n* = 28		*p* value
Depression-dejection (0–32)	4.8 ± 5.4 (0–19)	3.3 ± 5.2 (0–20)	0.102
Vigour-activity (0–24)	7.0 ± 5.6 (0–18)	8.5 ± 5.7 (0–20)	0.293
Anger-hostility (0–28)	3.9 ± 4.5 (0–18)	2.4 ± 2.9 (0–9)	0.134
Tension-anxiety (0–24)	4.1 ± 3.8 (0–13)	3.6 ± 4.3 (0–16)	0.351
Confusion-bewilderment (0–20)	2.1 ± 1.9 (0–8)	2.1 ± 3.0 (0–10)	0.324
Fatigue-inertia (0–20)	5.7 ± 4.9 (0–20)	5.6 ± 5.8 (0–21)	0.469
POMS Total (0–100)	13.7 ± 19.8 (14–59)	8.6 ± 18.4 (18–49)	0.306
Beck Depression Inventory (BDI)	*n* = 28	*n* = 28	
Level of depression (0–63)	11.8 ± 7.5 (1–35)	8.2 ± 6.6 (0–26)	0.037*

*Significant at <0.05 level

## Discussion

This study identified factors peculiar to patients with RA and persistent fatigue, despite a good disease response to 6-month TNF inhibitor therapy, by comparing those with persistent fatigue to those with no fatigue. There were no significant differences in key or minor characteristics between the groups at initiation of TNF inhibitor treatment, as reported elsewhere [8]. However, there were some statistically or clinically significant differences in group characteristics at 6 months post-initiation of TNF inhibitor therapy. The HAQ-DI did exceed the known minimal clinical important difference between groups even though this was not statistically significant [31]. This is an important observation, as functional health status is repeatedly reported to influence fatigue outcome in RA [6, 32]. The proportion of patients within the persistent fatigue group who were RF-positive was significantly greater than that in the no-fatigue group. The persistent fatigue group also reported significantly longer EMS duration; this finding is in keeping with a published longitudinal study which showed a significant association between stiffness on awakening and higher fatigue [33]. Furthermore, qualitative study has shown that patients regard the absence of stiffness as an important criterion for remission [34]. The lower incidence of ever-failing DMARDs found in the persistent fatigue group was contrary to what might have been expected.

Inclusion criteria for this study specified the demonstration of a moderate to good disease response to TNF inhibitor therapy, according to the ERC, as opposed to a specific DAS28 target. However, the mean DAS28 score (≈3.3) reflected a low disease activity state for patients with persistent fatigue. However, the statistically significant and even lower mean DAS28 score demonstrated in 93 % of those with no fatigue reflected a state of clinical remission (DAS28 <2.6) [17], versus only 37 % of the persistent fatigue group. These data also indicate an association between persistent fatigue and other unfavourable disease characteristics [35], including a positive RF [36]. Assertions within the literature suggest that DAS28 <2.6 is more representative of minimal disease activity than of remission [37]. Overall, these data show an association between persistent fatigue and an on-going state of low disease activity. It therefore highlights the need to look beyond disease remission for optimal disease management to improve symptom burden and patient outcomes.

With respect to the patient-reported outcome, pain findings confirm that in patients with disease severity which warranted TNF inhibitor therapy, those with persistent fatigue experienced more pain than those with no fatigue [3, 6, 38]. This interrelationship was previously reported from studies with either mixed diagnostic groups or non-biologic-treated populations [3, 9]. Further, the nature of this pain was reported to be more physical in nature as opposed to being affective/emotionally driven. Although the recent systematic review of fatigue and factors related to fatigue in RA found conflicting evidence with respect to the relationship between pain and fatigue, nonetheless, pain was one of three variables with the strongest evidence for a relationship with fatigue [6]; this study supports the dominant interrelationship between pain and fatigue. As patients in this study had demonstrated a good disease response to 6 months of TNF inhibitor treatment at time of assessment, these findings in a new treatment group add to the body of evidence of previous work illustrating that physical pain is a more important driver of persistent fatigue in RA than is inflammation [3, 38, 39]. This study result therefore underscores why proactive pain management should continue to be a priority in comprehensive patient care.

SE as a previously reported contributor to fatigue [6] was not substantiated by this study. In a previous systematic review, five studies, undertaken in largely non-biologic populations, supported SE and only one demonstrated no interrelationship between SE and fatigue [6]. Nonetheless, as in this current study, the systematic review failed to demonstrate that SE majorly influenced fatigue status in RA. This current lack of association may, in part, be explained by the superior clinical efficacy of biologic medications.

This study found that patients with persistent fatigue had significantly more depression than those with no fatigue. While published evidence is conflicting with respect to the contribution of depression towards the symptom of fatigue in RA; nonetheless, strong evidence of a relationship between these two symptoms exists [6]. While this association between fatigue and depression is recognised in RA [6], so too is the existence of a bidirectional causal pathway [40]. One previous study found a steep increase in depression as functional limitation increased [41], while another TNF inhibition study in RA showed that both fatigue and pain significantly impacted on changes in depression status and that clinical remission improved symptoms of depression [42]. There is a risk that the interrelationship between pain and fatigue [43] is minimised by some literature that highlights the mediating effect of mood disorders [6]. With respect to symptom management for this patient group, such an assumption could result in suboptimal pain and fatigue management. Moreover, physiological pain and depression, identified as problematic within the persistent fatigue group, are potential modifiable drivers of fatigue.

Similar patient numbers per subgroup used sleep-inducing medication, and both groups experienced a poor sleep quality. In keeping with previous work [44], this was attributed to physical discomforts, namely, that joint pain and limitation due to pain mediated the association between arthritis and insomnia [45]. However, findings from this study do not suggest any association between persistent post-treatment fatigue and sleep quality.

It is important to highlight study limitations inherent in a naturalistic study design. This study was conducted in a real-life clinical situation with an inability to address completeness of data and study attrition. Study replication sufficiently powered to support multiple regression analysis is recommended. Further, the comparative study of persistent post-treatment fatigue was restricted to the single disease entity of RA. While a high response rate was achieved, the generalisation of results to non-RA patients is not appropriate. It is also acknowledged that the uniqueness of a single-site academic centre which had poor representation of minority groups restricts the representativeness of findings. Data on possible confounding variables such as daily physical activities, physical exercise capacity, thyroid function and morning (08.00 h) adrenocorticotropic hormone (ACTH)/cortisol values was not collected. This comparative study adds further to the body of knowledge on factors related to persistent fatigue in patients with RA [6, 7] and to the limited body of knowledge with respect to fatigue in TNF inhibitor-treated patients [3, 32]. It firmly highlights potential modifiable factors to improve fatigue, namely, pain and depression. In addition, it highlights the association between persistent fatigue and an on-going state of low disease activity. This infers that more judicious disease management could minimise the symptom burden of pain and depression and consequentially fatigue.
